# Pharmacokinetics of desflurane uptake and disposition in piglets

**DOI:** 10.3389/fphar.2024.1339690

**Published:** 2024-04-02

**Authors:** Chih-Cherng Lu, Shung-Tai Ho, Oliver Yao-Pu Hu, Cheng-Huei Hsiong, Yuan-Chen Cheng, Che-Hao Hsu, Tso-Chou Lin

**Affiliations:** ^1^ Department of Anesthesiology, Tri-Service General Hospital, National Defense Medical Center, Taipei, Taiwan; ^2^ Department of Anesthesiology, Taipei Veterans General Hospital, National Defense Medical Center, Taipei, Taiwan; ^3^ Department of Anesthesiology, Kaohsiung Medical University Hospital, Kaohsiung Medical University, Kaohsiung, Taiwan; ^4^ School of Pharmacy, National Defense Medical Center, Taipei, Taiwan; ^5^ Internship, E-Da Hospital, I-Shou University College of Medicine, Kaohsiung, Taiwan; ^6^ Department of Anesthesiology, Tungs’ Taichung MetroHarbor Hospital, Taichung, Taiwan

**Keywords:** inhalational anesthetic, desflurane, pharmacokinetic, uptake, disposition, piglet

## Abstract

**Introduction:**

Many respiratory but few arterial blood pharmacokinetics of desflurane uptake and disposition have been investigated. We explored the pharmacokinetic parameters in piglets by comparing inspiratory, end-tidal, arterial blood, and mixed venous blood concentrations of desflurane.

**Methods:**

Seven piglets were administered inspiratory 6% desflurane by inhalation over 2 h, followed by a 2-h disposition phase. Inspiratory and end-tidal concentrations were detected using an infrared analyzer. Femoral arterial blood and pulmonary artery mixed venous blood were sampled to determine desflurane concentrations by gas chromatography at 1, 3, 5, 10, 20, 30, 40, 50, 60, 80, 100, and 120 min during each uptake and disposition phase. Respiratory and hemodynamic parameters were measured simultaneously. Body uptake and disposition rates were calculated by multiplying the difference between the arterial and pulmonary artery blood concentrations by the cardiac output.

**Results:**

The rates of desflurane body uptake increased considerably in the initial 5 min (79.8 ml.min^−1^) and then declined slowly until 120 min (27.0 ml.min^−1^). Similar characteristics of washout were noted during the subsequent disposition phase. Concentration–time curves of end-tidal, arterial, and pulmonary artery blood concentrations fitted well to zero-order input and first-order disposition kinetics. Arterial and pulmonary artery blood concentrations were best fitted using a two-compartment model. After 2 h, only 21.9% of the desflurane administered had been eliminated from the body.

**Conclusion:**

Under a fixed inspiratory concentration, desflurane body uptake in piglets corresponded to constant zero-order infusion, and the 2-h disposition pattern followed first-order kinetics and best fitted to a two-compartment model.

## Highlights

1. Many human and animal studies have explored pharmacokinetics of desflurane anesthesia by using inspiratory and expiratory concentrations, but with scarce arterial blood data.
2. This piglet study administered inspiratory 6% desflurane for 2-h uptake phase and then discontinuation for 2-h disposition phase by comparing the end-tidal, arterial, and pulmonary artery blood concentrations.
3. Concentration–time curves fitted well to zero-order input and first-order disposition kinetics.
4. At 5 min after discontinuation, the end-tidal and arterial blood concentrations were similar with those in our previous human study.
5. After 2 h disposition, only 21.9% of the desflurane administered had been eliminated from the body.

## 1 Introduction

Desflurane, an inhalational anesthetic introduced into clinical practice in the 1990s, possesses lower blood and fat tissue solubilities than sevoflurane or isoflurane, thus enabling rapid equilibration and elimination (Eger, 1995; Jakobsson, 2012; Esper et al., 2015), especially in patients with overweight (Singh et al., 2017) and after prolonged anesthesia (Nordmann et al., 2006; Lin et al., 2013). Following the cessation of administration, desflurane has a predictable emergence in approximately 5 min (Lin et al., 2013; Werner et al., 2015). Patients using desflurane have an earlier extubation time than those with sevoflurane (Dexter et al., 2010), isoflurane (Agoliati et al., 2010), or propofol (Wachtel et al., 2011).

To date, no clinically pharmacodynamic reversal agents are available for inhalational anesthetics. Instead, recovery of consciousness is largely dependent on pharmacokinetic processes leading to drug elimination via respiratory exchange (Vincent, 2023). Using inspiratory and end-tidal concentrations, the respiratory pharmacokinetic parameters of desflurane have been experimentally assessed in pigs (30-min uptake) (Yasuda et al., 1990), horses (2-h uptake) (Valente et al., 2015), healthy volunteers (30-min and 8-h uptake) (Yasuda et al., 1991; Eger et al., 1997), and in the simulation models during elimination (Lockwood, 2010; Weber et al., 2021). In addition, hyperventilation or a high ventilation/perfusion ratio can facilitate desflurane wash-in (Lu et al., 2012) and washout (Kretzschmar et al., 2021). Under stable lung ventilation and cardiac output, a two-compartment model accurately captured desflurane elimination in patients in terms of actual inspired and end-expired concentrations (Wissing et al., 2000), as well as those concentrations by a simulation model (Rietbrock et al., 2000).

Arterial blood delivers inhalation anesthetics to the brain, the site influencing the depth of anesthesia. Arterial concentrations more closely reflect those in the brain compared to end-tidal concentrations. However, only few studies demonstrated the arterial blood pharmacokinetics of desflurane in piglets during 45-min uptake followed by 45-min elimination (Kretzschmar et al., 2021) and in patients during 1-h uptake (Lu et al., 2004), 2-h hypothermic cardiopulmonary bypass (Mets et al., 2001), and 20-min elimination after surgery (Lin et al., 2013; Lu et al., 2013). Clinically, human studies on the prolonged elimination of desflurane are not feasible because of the rapid emergence and short extubation time (<20 min) of the anesthetic (Dexter et al., 2010). Therefore, in this study, we explored the 2-h uptake and 2-h disposition kinetics of desflurane in piglets by comparing the inspiratory and end-tidal concentrations with the systemic arterial blood and pulmonary artery mixed venous blood concentrations.

## 2 Material and methods

### 2.1 Ethics approval

The Institutional Animal Care and Use Committee of the National Defense Medical Center in Taipei, Taiwan approved the study protocol (NDMC-IACUC-08-028). The care and use of the animals were in full compliance with the guidelines of the institution and the approved protocol.

### 2.2 Animals

Seven domestic 5-month-old male piglets weighing 28 ± 5 kg on average were used in the study. The piglets fasted overnight with free access to water. The experiments were conducted between 8:00 a.m. and 3:00 p.m. All piglets underwent the same protocol (monitoring and anesthesia induction and maintenance).

### 2.3 Anesthetic management

General anesthesia was induced with intramuscular tiletamine/zolazepam (6 mg.kg^−1^; Zoletil; Virbac, France) and atropine (0.01 mg.kg^−1^; Taiwan Biotech Co., Taiwan), supplemented with a continuous infusion of fentanyl (Pharmaceutical Plant of Controlled Drugs of Taiwan Food and Drug Administration, Taiwan) and propofol (Anesvan; Chi-Sheng, Taiwan) at 10–20 μg.kg^−1^.h^−1^ and 5–10 mg.kg^−1^.h^−1^, respectively, via an auricular vein. After tracheostomy in the supine position, muscle relaxation was maintained with an intravenous bolus of 0.15 mg.kg^−1^ pancuronium (Pavulon; Organon, Netherland) and followed by a continuous infusion of 0.04–0.08 mg.kg^−1^.h^−1^. The lungs were mechanically ventilated with 40% oxygen by using a conventional piston-pump ventilator (Harvard Large Animal Volume Controlled Ventilator, model 613) with a tidal volume of 8 ml.kg^−1^ and a positive end-expiratory pressure of 5 cmH_2_O. The respiratory rate was adjusted to maintain the end-tidal CO_2_ partial pressure within 35–45 mmHg.

To sample mixed venous blood, an Opti-Q catheter (Abbott Critical Care Systems, Mountain View, CA, United States) was placed into the pulmonary artery through an external jugular vein. The catheter was linked to the Q-Vue Continuous Cardiac Output Computer (Abbott Critical Care Systems). Calibrated through thermodilution, iced saline was applied in triplicate every 20 min. A 20-gauge femoral arterial catheter was inserted for continuous arterial pressure measurement and for blood sampling. After 30 min of stabilization, the infusions of fentanyl and propofol infusion were stopped. At 5 l.min^−1^ gas flow, 40% oxygen was passed through an anesthetic machine with a desflurane vaporizer delivering 6% desflurane without soda lime. The entire circuit was prewashed for 5 min with desflurane at the minimum alveolar concentration for pigs (6%). Next, the 6% desflurane was fed into a Harvard animal ventilator for 2 h. Following a 2-h uptake phase, 40% oxygen was fed directly into the Harvard animal ventilator at 5 l.min^−1^ for another 2 h (i.e., the disposition phase). Anesthesia was maintained using an infusion of fentanyl and propofol at 5–10 μg.kg^−1^.h^−1^ and 5–10 mg.kg^−1^. h^−1^, respectively. Hypotension, defined as a 25% reduction in blood pressure from baseline, was treated with 5 mg of ephedrine through an intravenous bolus injection.

The inspiratory and end-tidal concentrations of desflurane, along with the end-tidal carbon dioxide concentration, blood pressure, heart rate, and cardiac output, were recorded continuously throughout the study. Furthermore, the piglets’ body temperature was maintained at approximately 37°C by using heated blankets, as monitored using the Opti-Q catheter. At the end of the study, the piglets were euthanized with an intravenous injection of pentobarbital (100 mg kg^-1^; Easy Go; Health-Tech Pharmaceutical Co., Taiwan) and then cessation of ventilation.

### 2.4 Determination of inspiratory and end-tidal desflurane concentrations (partial pressure)

Volatile anesthetic concentrations (percentages) were monitored using a multigas analyzer (Datex-Ohmeda S/5 Compact Anesthesia system; Datex, Helsinki, Finland). The analyzer was calibrated prior to each experiment according to the manufacturer’s instructions. End-tidal concentrations below 0.2% of desflurane were discarded due to the lowest limit of detection accuracy.

### 2.5 Blood sampling

Before desflurane administration, 20 ml of arterial blood was drawn from each piglet through the femoral artery catheter to determine the blood–gas partition coefficient (λ) and thus construct individual blood calibration curves. Next, 1, 3, 5, 10, 20, 30, 40, 50, 60, 80, 100, and 120 min into each uptake and disposition phase, 1-ml aliquots of blood were collected simultaneously from the femoral artery and pulmonary artery catheters by using heparinized syringes. Each sample was immediately placed into a 10-ml glass vial, which was then tightly sealed. The samples were stored at 0°C and measured within 24 h. All samples were analyzed for desflurane concentrations by gas chromatography by using a headspace sampler and a flame ionization detector under the methods detailed in the previous studies (Lu et al., 2004; Peyton et al., 2007; Lu et al., 2012; Lin et al., 2013; Lu et al., 2013) (Supplementary Material).

### 2.6 Body uptake and pulmonary disposition of desflurane

The desflurane vapor content in the arterial and pulmonary artery blood samples is expressed in milliliters of desflurane. The rates of body uptake and disposition of desflurane vapor, in milliliters per minute, were calculated by taking the percentage differences between the concentrations of blood from the femoral and pulmonary arteries (ml.dl^−1^) and multiplying the result by the cardiac output (l.min^−1^) and then by 10. The total amounts of desflurane vapor during body uptake and disposition were estimated from the area under the uptake time and disposition time curves of arterial and pulmonary artery blood concentrations. The amount of desflurane vapor deriving from 1 ml of desflurane fluid is 210 ml by Henry’s law (Biro, 2014). The ratio of disposed over uptake amounts of desflurane fluid in blood (ml) were calculated accordingly.

### 2.7 Calculation of pharmacokinetic parameters

WinNonlin 5.2, a pharmacokinetic program (Pharsight Corporation, Mountain View, CA, United States), was used to fit the data to monoexponential, biexponential, and triexponential models to determine the pharmacokinetic parameters. The Akaike information criterion was used to determine the goodness of fit of each desflurane concentration–time curve (Yamaoka et al., 1978). Regarding desflurane disposition, the pharmacokinetic parameters, namely, volume distribution, clearance, disposition rate, disposition half-life, area under the concentration–time curve, area under the moment curve, and mean residence time, were all estimated from the model interpretation. Both the desflurane concentrations and estimated pharmacokinetic parameters are reported as means (standard deviations).

### 2.8 Statistical analysis

Analyses were conducted using IBM SPSS Statistics for Windows, version 22 (IBM Corp., Armonk, NY, United States). The descriptive statistics of hemodynamic and ventilatory variables are expressed as means (standard deviations). The significance of any difference in parametric values was assessed through one-way analysis of variance followed by multiple comparisons with Scheffé’s method. *p* values of <0.05 were considered significant.

## 3 Results

The blood–gas partition coefficient of desflurane before administration was 0.38 ± 0.01. As presented in Table 1, the desflurane vapor concentrations following the initiation of desflurane administration can be ordered in descending order as follows: inspiratory > end-tidal > femoral artery blood > pulmonary artery mixed venous blood concentrations. During the uptake phase, the average ratios of arterial over end-tidal concentrations ranged between 0.77 and 0.82. This step-down pattern was reversed during the disposition phase, during which the average ratios of arterial blood to pulmonary artery blood concentrations ranged between 0.64 and 0.74. The uptake rate of desflurane vapor concentration peaked to 79.8 ± 24.8 ml.min^−1^ at 5 min and then declined constantly to 27.0 ± 15.9 ml.min^−1^ at 120 min. The disposition rates decreased rapidly from 47.0 ± 17.9 ml.min^−1^ at 1 min to 26.6 ± 12.0 ml.min^−1^ at 5 min. The total body uptake (determined from the difference in concentrations between the arterial and mixed venous blood) over 2 h was 23.2 ± 6.7 ml desflurane fluid by blood and tissue. The total amount of desflurane eliminated over 2 h (determined from the difference in concentration between the mixed venous and arterial blood) was only 5.1 ± 0.9 ml (21.9%).

**TABLE 1:** Inspiratory, end-tidal, arterial blood, and pulmonary artery blood concentrations of desflurane and rates of body uptake and disposition.

	Uptake phase (min)							Disposition phase (min)						
Concentration	1	3	5	10	20	60	120	1	3	5	10	20	60	120
Inspiratory, %	5.41 (0.49)	5.93 (0.48)	5.74 (0.34)	6.04 (0.14)	6.04 (0.10)	6.00 (0.04)	5.99 (0.03)	0	0	0	0	0	0	0
End-tidal (ET), %	3.39 (0.79)	4.75 (0.62)	4.93 (0.23)	5.35 (0.28)	5.45 (0.09)	5.61 (0.09)	5.76 (0.05)	2.18 (0.88)	1.23 (0.47)	1.04 (0.55)	0.64 (0.17)	0.38 (0.10)	0.20 (0.04)	--
Arterial blood (A), %	2.75 (0.67)	3.65 (0.39)	4.00 (0.53)	4.17 (0.54)	4.44 (0.39)	4.58 (0.40)	4.65 (0.26)	2.23 (0.87)	1.39 (0.43)	1.24 (0.47)	0.84 (0.17)	0.54 (0.07)	0.32 (0.05)	0.23 (0.06)
Pulmonary artery blood (PA), %	1.15 (0.65)	2.24 (0.45)	2.61 (0.39)	3.02 (0.26)	3.27 (0.34)	3.62 (0.47)	4.10 (0.41)	2.96 (0.54)	2.08 (0.39)	1.69 (0.33)	1.22 (0.23)	0.85 (0.13)	0.49 (0.06)	0.34 (0.05)
A/ET during uptake	0.82 (0.15)	0.77 (0.08)	0.81 (0.12)	0.78 (0.09)	0.82 (0.07)	0.82 (0.07)	0.81 (0.05)	--	--	--	--	--	--	--
PA/A during uptake	0.41 (0.20)	0.61 (0.09)	0.66 (0.06)	0.73 (0.06)	0.74 (0.04)	0.79 (0.05)	0.88 (0.06)	--	--	--	--	--	--	--
ET/A during disposition	--	--	--	--	--	--	--	0.98 (0.12)	0.87 (0.07)	0.82 (0.09)	0.76 (0.12)	0.70 (0.13)	0.65 (0.22)	--
A/PA during disposition	--	--	--	--	--	--	--	0.74 (0.18)	0.67 (0.12)	0.72 (0.14)	0.69 (0.10)	0.64 (0.09)	0.66 (0.07)	0.68 (0.12)
Cardiac output, l.min^-1^	3.8 (1.5)	3.8 (1.3)	4.7 (1.9)	3.0 (1.5)	3.9 (1.5)	4.0 (1.1)	5.1 (1.7)	5.8 (1.3)	5.8 (1.3)	5.8 (1.2)	4.7 (1.5)	4.7 (1.6)	3.8 (1.6)	4.1 (2.0)
*Rates of body uptake/disposition in vapor, ml.min^-1^	61.3 (29.3)	64.1 (24.8)	79.8 (24.8)	34.5 (20.6)	44.7 (16.7)	36.7 (4.9)	27.0 (15.9)	47.0 (17.9)	36.1 (13.1)	26.6 (12.0)	19.5 (5.0)	14.1 (5.5)	5.8 (1.2)	4.0 (1.4)

Data are presented as means (standard deviations).

A, arterial blood concentration, % in vapor; ET, end-tidal concentration, % in vapor; PA, pulmonary artery blood concentration, % in vapor.

*Rate of body uptake in vapor [ml.min^-1^] = 10 x (A− PA) [ml.dl^-1^] × cardiac output [l.min^-1^].

Rate of body disposition in vapor [ml.min^-1^] = 10 × (PA − A) [ml.dl^-1^] × cardiac output [l.min^-1^].

The estimated total uptake and disposition of desflurane fluid in blood was 23.2 ± 6.7 ml and 5.1 ± 0.9 ml, respectively, by Henry’s law (Biro, 2014).

**FIGURE 1:**
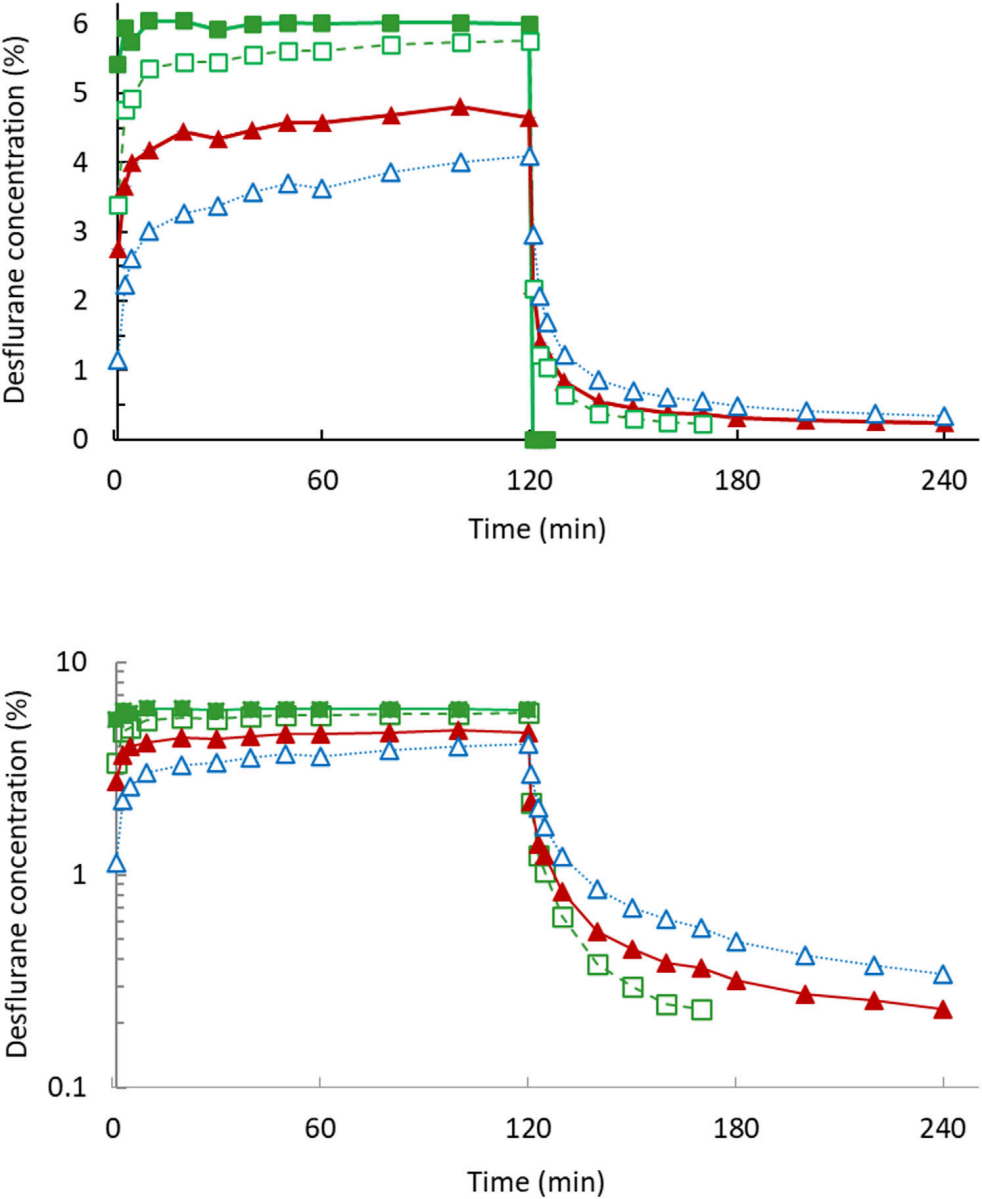
Desflurane mean concentration–time curves in 2-h uptake and 2-h disposition phases (upper). The semi-log plot (lower) dipictdes constant zero-order infusion during uptake and first-order kinetics during disposition phase. Inspiratory concentration (filled square); end-tidal concentration (open square); arterial blood concentration (filled triangle); pulmonary artery mixed venous blood concentration (open triangle).

**FIGURE 2:**
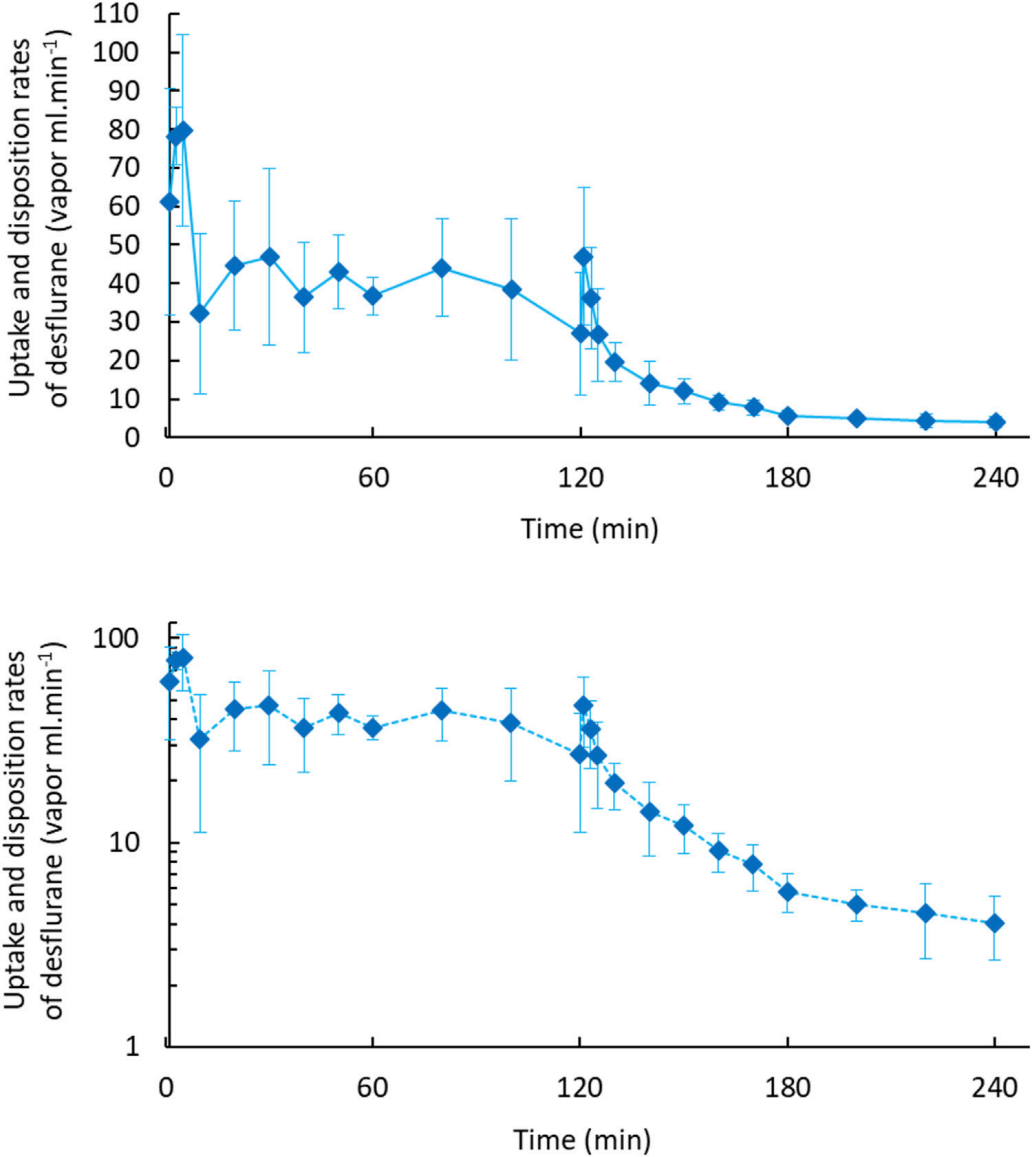
Body uptake and disposition rates of desflurane vapour in 2-h uptake and 2-h disposition phases (upper). The semi-log plot (lower) dipictdes constant zero-order infusion during body uptake and first-order kinetics during the disposition phase.

Figure 1 displays the concentration–time curves and an exponential decline with a constant first-order disposition rate on a semilogarithmic plot in 2 h. Figure 2 depicts the rates of body uptake and disposition of desflurane vapor.

The hemodynamic and respiratory variables, namely, heart rate, mean arterial pressure, minute ventilation, and end-tidal carbon dioxide levels, are summarized in Table 2. No significant differences throughout the uptake or disposition phases were observed.

**TABLE 2:** Hemodynamic and respiratory variables.

	Uptake phase (min)							Disposition phase (min)						
	1	3	5	10	20	60	120	1	3	5	10	20	60	120
Heart rate, beats per min	116 (27)	118 (29)	115 (29)	111 (29)	114 (29)	127 (24)	141 (31)	136 (35)	133 (35)	133 (35)	131 (32)	129 (30)	104 (36)	107 (39)
Mean arterial pressure, mmHg	105 (26)	96 (19)	91 (21)	86 (41)	85 (14)	90 (16)	108 (20)	115 (19)	128 (24)	126 (17)	138 (17)	126 (20)	98 (18)	100 (28)
ETCO_2_, mmHg	37 (6)	36 (4)	35 (4)	37 (5)	37 (5)	40 (4)	40 (2)	40 (3)	40 (3)	43 (6)	39 (3)	39 (3)	39 (4)	42 (3)
Tidal volume, ml	346 (43)	323 (39)	322 (38)	303 (50)	296 (51)	279 (49)	299 (45)	316 (69)	314 (64)	297 (100)	354 (62)	331 (50)	314 (40)	304 (47)
Respiratory rate, per min	9.3 (1.5)	9.4 (1.1)	9.9 (0.4)	9.9 (0.4)	9.9 (0.4)	10.0 (0)	10.1 (0.4)	10 (0)	10 (0)	10 (0)	10 (0)	10 (0)	10 (0)	10 (0)
Minute ventilation, ml.min^-1^	3.2 (0.5)	3.0 (0.5)	3.2 (0.4)	3.0 (0.5)	2.9 (0.5)	2.8 (0.5)	3.0 (0.4)	3.2 (0.7)	3.1 (0.6)	3.0 (1.0)	3.5 (0.6)	3.3 (0.5)	3.1 (0.4)	2.6 (1.1)

Values are presented as means (standard deviations).

ETCO_2_, end-tidal carbon dioxide concentration.

The estimated pharmacokinetic parameters of the end-tidal, femoral artery blood, and pulmonary artery blood concentrations are shown in Table 3. The data fitted well to a biexponential curve for arterial blood and pulmonary artery blood concentration–time curves. The clearance derived from both concentration–time curves were comparable. The alpha and beta half-lives, distribution volume of steady state, and mean residence time of the pulmonary artery blood were all significantly greater than those of the arterial blood.

**TABLE 3:** Pharmacokinetic parameters of the end-tidal, arterial blood, and pulmonary artery blood concentrations during disposition phase.

Parameters	End-tidal concentration	Arterial blood concentration	Pulmonary artery blood concentration
A (%)	731.26 (395.62)	514.32 (149.64)^a^	147.32 (58.32)
B (%)	3.45 (1.71)	2.11 (0.70)	2.10 (0.50)
Alpha half-life, min	0.75 (0.51)	0.71 (0.31)^b^	1.86 (0.57)
Beta half-life, min	61.90 (105.93)	46.39 (14.43)^c^	61.29 (15.35)
Distribution volume of central compartment, l	0.10 (0.09)	1.09 (0.42)^b^	0.35 (0.14)
Distribution volume of steady state, l	1.29 (2.36)	1.20 (0.44)^a^	2.75 (0.94)
Clearance, l.min^-1^	0.07 (0.02)	0.08 (0.02)^a^	0.09 (0.02)
Mean residence time, min	15.51 (24.98)	15.00 (3.39)^b^	31.26 (8.96)
Maximal concentration, %	5.68 (0.40)	4.85 (0.42)^a^	4.07 (0.29)
Area under the curve, % × min	695.88 (27.73)	603.03 (50.65)^a^	533.10 (30.47)

Values are presented as means (± standard deviations).

Biexponential equation: *Y* = *A* × exp (−Alpha × Time) + *B* × exp (−Beta × Time), where *Y* represents the end-tidal, arterial blood, and pulmonary artery blood concentrations. a, *p* < 0.001; b, *p* < 0.01; c, *p* < 0.05, compared with the significance levels of the pulmonary artery blood concentrations.

**FIGURE 3:**
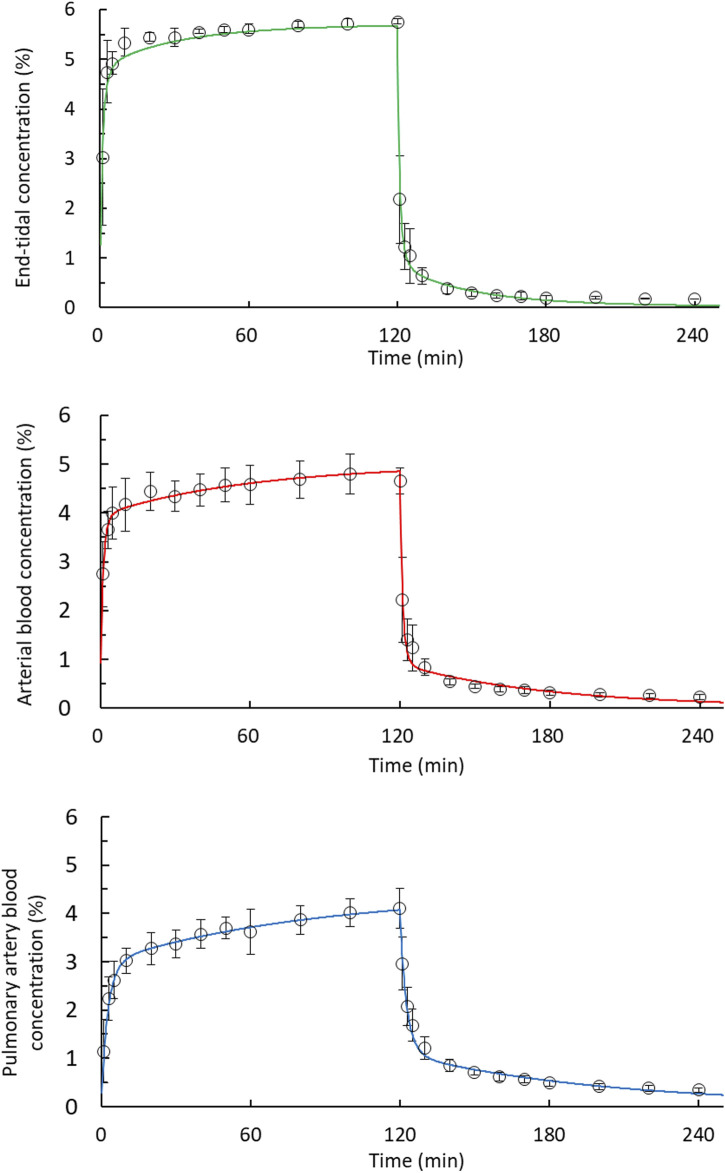
Fit of the desflurane concentration–time curves corresponded to zero-order input and to a first-order disposition kinetics model in 2-h uptake and 2-h disposition phases. The end-tidal, arterial blood, and pulmonary artery blood concentrations were fitted using a two-compartment model during the disposition phase. Observed concentrations (open circles); predicted concentrations (solid line).

In Figure 3, the end-tidal, arterial blood and pulmonary artery blood concentration–time curves fitted well to zero-order input and first-order disposition kinetics.

## 4 Discussion

### 4.1 Major findings

This is the 2-h pharmacokinetic disposition study of desflurane in piglets following a 2-h uptake phase. Notably, the end-tidal, arterial, and pulmonary concentration–time curves all fitted well to zero-order input (intravenous constant infusion model) during the uptake phase and to first-order disposition kinetics. We observed an initial fast 5-min distribution phase during the uptake phase, indicating a dilution effect attributable to the functional residual capacity (FRC). The arterial blood and pulmonary artery blood concentrations were fitted by a two-compartment model during the disposition phase. At the end of that phase, near 80% of the administered desflurane remained in the body despite the fact that the concentration of desflurane in the femoral artery and pulmonary artery blood had decreased by almost 90%.

### 4.2 Constant body uptake with zero-order input

The uptake and elimination of inhalational anesthetics go through four main parts: pulmonary alveoli, vessel-rich organs (the brain), skeletal muscles, and fat tissues. In the first 3 min of wash-in, FRC acts as dead space or as an extension of the anesthetic circuit (Lin, 1994). A greater inspiratory–expiratory difference due to the dilution of the initial inspiratory concentration by gases pre-existing in the lungs raises the rate of lung uptake (Hendrickx et al., 2016), but also limits the amount of body uptake. After FRC wash-in, the alveolar (end-tidal) concentration reaches the level of the given anesthetic concentration (Lin, 1994; Hendrickx et al., 2016). Regarding the attainment of the targeted anesthesia depth in the brain, subsequent uptake across the alveolar membrane and blood–brain barrier depends on the partition coefficients (Hsu et al., 2021) and cardiac output, (Kretzschmar et al., 2021), indicating the “hysteresis” phenomenon as the delay between changes in end-tidal partial pressure and changes in the target tissue, the brain (Hendrickx and De Wolf, 2022). Lu et al. observed that under a fixed inspiratory concentration and stable cardiopulmonary parameters, the uptake of desflurane was relatively constant (arterial end-tidal ratio: 0.69 to 0.73 at 30–60 min) in cardiac patients (Lu et al., 2004). In the present study, the ratios of arterial over end-tidal concentrations were comparable (between 0.77 and 0.82) during the 2-h uptake phase, indicating a fixed blood/gas partition coefficient across the alveolar membrane. We also noted a relatively constant body uptake after lung wash-in, with ratios of pulmonary artery over arterial blood concentrations of 0.73–0.88. The end-tidal, arterial, and pulmonary artery concentration–time curves were all well fitted with zero-order absorption when compared with intravenous infusion anesthesia models.

### 4.3 Rapid emergence and ET concentrations at 5 min after FRC washout

Without clinically available pharmacodynamic reversal agents for inhaled anesthetics, recovery of consciousness is largely dependent on respiratory pharmacokinetic elimination (Vincent, 2023). Consequently, delayed emergence, emergency delirium, and associated higher costs in the operating room have been under active investigation. Immediately after the cessation of desflurane administration, alveolar concentrations were rapidly diluted with 40% oxygen fresh gas. Within the first minute, arterial concentrations fell steeply below pulmonary artery concentrations, creating the largest gradients between pulmonary artery, arterial, and end-tidal concentrations. In human studies, within the initial 5 min of oxygen supply (6 l.min^−1^), rapid lung clearance through FRC washout was noted (Lin et al., 2013; Lu et al., 2013), with a rapid washout of arterial concentration due to a low blood/gas partition coefficient of 0.45–0.57 for desflurane (Jakobsson, 2012; Esper et al., 2015). In two studies, the awakening or emergence time, defined as the time between desflurane discontinuation to the observation of an eye opening response to a verbal command, was 5.0 ± 2.5 min after urological cystoscopic surgery (Werner et al., 2015) and 5.2 ± 1.6 min after gynecological surgery (Lin et al., 2013). The mean end-tidal and arterial blood concentrations on awakening (0.96% and 1.20%, respectively) in the gynecological patients (Lin et al., 2013) had favorable consistency with the corresponding concentrations (1.04% and 1.24%, respectively) at 5 min in the current study. After emergence from desflurane anesthesia, subsequent human elimination study is not clinically feasible. Therefore, we conducted an extended pharmacokinetic study of desflurane in piglets by using a propofol infusion.

### 4.4 Two-compartment pharmacokinetic model of desflurane disposition

Under stable hemodynamic parameters, two distinct disposition components, a rapid 5-min component followed by a slow 15-min component, have been described in human studies (Wissing et al., 2000; Lu et al., 2013) and a derived simulation study (Rietbrock et al., 2000). In a study on juvenile pigs, after 5 min of desflurane washout, the ratio of anesthetic partial pressure in arterial to mixed venous blood reached a constant value (Kretzschmar et al., 2021), indicating body washout. This is consistent with the range of 0.64–0.69 that we obtained in the present study. Therefore, the following disposition is dependent on the tissue/blood partition coefficients. Desflurane has a negligible body metabolism (0.02%) (Eger, 1995; Gouju and Legeay, 2023) and a lower fat/blood partition coefficient than sevoflurane (15 vs. 41) (Jakobsson, 2012). In the current study, the arterial and pulmonary artery concentrations dropped to one-tenth after the 2-h disposition phase. In a study by Lockwood (Lockwood, 2010), despite the rapid elimination of desflurane, a large amount is absorbed, and the amount of time taken for 99.9% of the anesthetic to be eliminated from the brain was 33 h. Moreover, 13% of the absorbed dose was retained in the body according to theoretical context-sensitive elimination (Lockwood, 2010). In our study, only 21.9% of the amount of desflurane absorbed over 2 h was eliminated over 2 h, as indicated by the uptake time and disposition time curves of desflurane concentrations in arterial and pulmonary artery blood. The residual effects of subanesthetic concentrations of desflurane following prolonged anesthesia should be considered.

### 4.5 Limitations

This study has two limitations. First, the end-tidal values fell below the detection limit of the multigas analyzer at 60 min after the cessation of desflurane administration. Therefore, the estimated pharmacokinetic parameters corresponding to end-tidal concentrations after 60-min disposition should be interpreted with caution. With a substantially lower detection limit, gas chromatography can yield more accurate pharmacokinetic measurements of the disposition phase, albeit clinically nonsignificant ones after emergence and extubation. Second, no filters were applied in the present study. Breathing circuit filters or combined heat- and moisture-exchanging filters are commonly employed for general anesthesia in clinical settings. However, Park et al. reported that a viral/bacterial filter added at the Y-piece of the breathing circuit or on the expiratory limb reduced the peak end-tidal concentration of desflurane (6.7% vs. 8.5%) compared with that the corresponding concentration obtained without filters (9.8%) after the inhalation of 10% desflurane over 5 min (Park et al., 2015). In summary, clinical end-tidal concentrations of desflurane might be affected by the use or nonuse of a circuit filter.

## 5 Conclusion

We have demonstrated that under a fixed inspiratory concentration, the body uptake rate of desflurane was similar to that under constant zero-order infusion, and the pattern of disposition obeyed first-order kinetics. The concentration–time curves of arterial blood and mixed venous blood were parallel, and the pharmacokinetic properties were comparable. At the end of the 2-h disposition phase, most of the desflurane administered remained in the body despite low end-tidal concentrations.

## Data Availability

The raw data supporting the conclusion of this article will be made available by the authors, without undue reservation.
