# Supplementary figures and images for: Pharmacokinetics of desflurane uptake and disposition in piglets
Source: Front Pharmacol. 2024 Apr 2;15:1339690. doi: 10.3389/fphar.2024.1339690 (PMC11018996; doi:10.3389/fphar.2024.1339690)

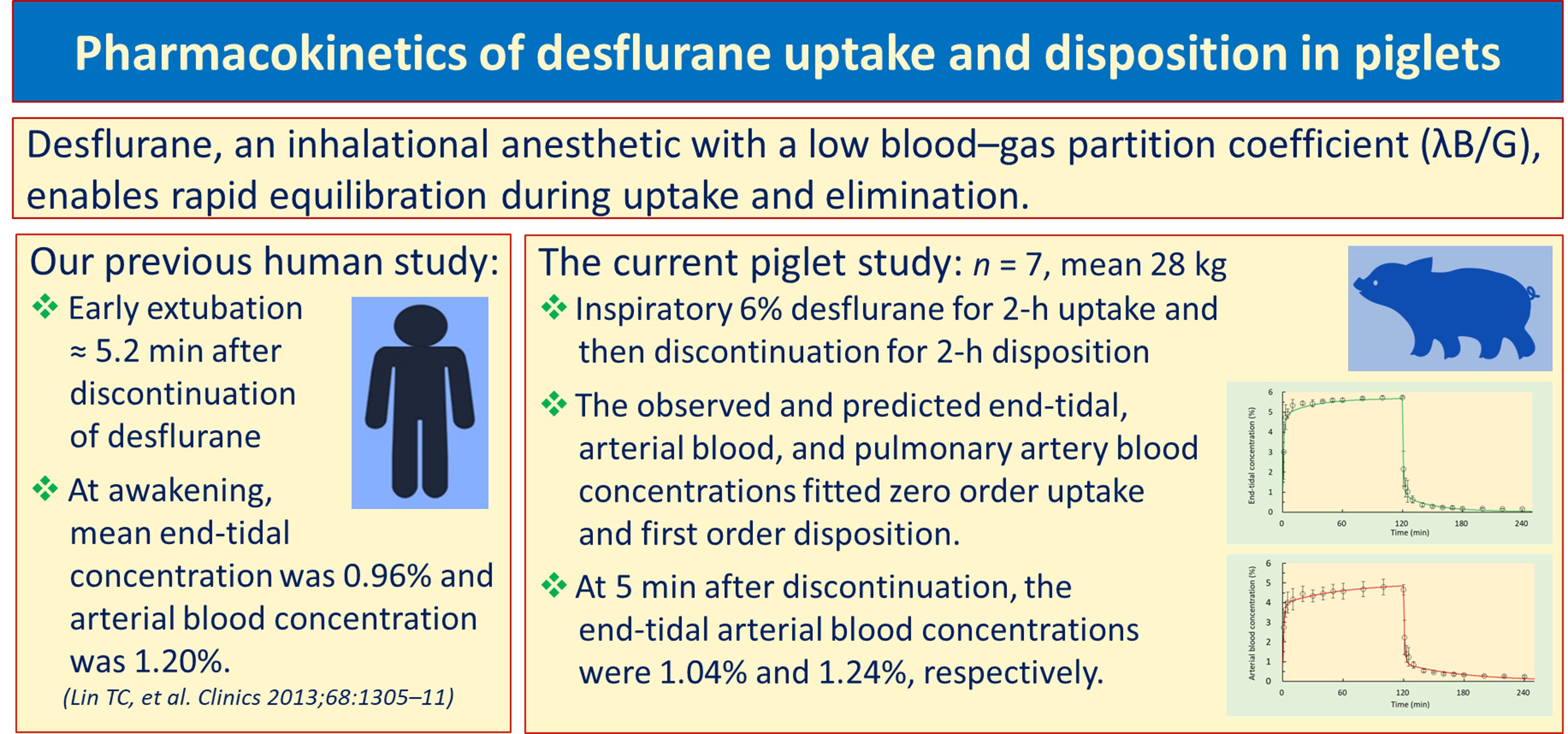

Supplement: Supplementary file 1 [file Image1.JPEG]
