# Supplementary material for: Pharmacokinetics of desflurane uptake and disposition in piglets
Source: Front Pharmacol. 2024 Apr 2;15:1339690. doi: 10.3389/fphar.2024.1339690 (PMC11018996; doi:10.3389/fphar.2024.1339690)
Supplement: Supplementary file 2 [file DataSheet1.PDF]

Supplementary Material

Appendix

I. Gas chromatography conditions for desflurane quantification

The HP 6890 series gas chromatography system (Hewlett-Packard, Wilmington, DE, USA) consisted of a Headspace Sampler (HP 7694 E), an oven, a flame ionization detector, and an integrator. The oven temperature was raised from 40ºC to 200ºC at a rate of 15ºC/min, held for 4.33 minutes, over a 15-minute cycle. Both injection and detection temperatures were set at 250ºC with the inlet pressure of 95 kPa. Injection was performed in the splitless mode, with 5.0-min purge off time. The carrier gas (nitrogen) flow rate was 3.0 mL/min, and separation was achieved using a capillary column (HP-5; 30.0 m × 0.32 mm internal diameter, 0.25 µm film thickness; Restek, Bellefonte, PA, USA). An integrator and a datum acquisition system were provided by HP Chemstation Software (Agilent Technologies, Santa Clara, CA, USA).

II. Primary calibration curve with known concentrations of desflurane gas:

The calibration curve was obtained by injecting different concentrations of desflurane into the gas chromatogram using a gas-tight Hamilton syringe. To obtain standard gas concentrations, five 550 mL glass bottles, each with a turnable stopcock and Teflon septum, were used. After flushing each bottle with nitrogen for 6 minutes, the septum was pierced with a syringe needle and 2, 5, 10, 20, or 50 µL of liquid desflurane were injected into the glass bottles at 4°C. The calculated volume percent of desflurane was plotted against the measured gas chromatogram counts.

III. Determination of desflurane blood / gas partition coefficients (λb/g) for each pig:

The blood/gas partition coefficients for desflurane can be calculated if concentrations of desflurane in the equilibrated sample headspace were determined by using the primary calibration curve. The blood/gas partition coefficients of desflurane were determined by the two-stage method as follows:

A 2-mL blood sample with unknown desflurane concentration was added into the first 10-mL vial (with 2 mL of gas removed before injection), sealed with a Teflon septum and then incubated at 37°C for 30 minutes. The desflurane concentration in the headspace of the first vial was determined by gas chromatography. One mL of sample was withdrawn immediately from the first vial and transferred to the second 10-mL vial (with one mL of gas removed before injection) sealed with a Teflon septum and then incubated at 37°C for another 30 minutes. The desflurane concentration in the headspace of the second vial was again determined by gas chromatography. Thus, the λ can be calculated by the equation in the following section.

IV. Calculation of desflurane contents in gas phase, in blood phase by known desflurane blood gas partition coefficients (λ):

According to the physical properties of desflurane,

MW / D = volume for on mole of desflurane liquid;

MW = molecular weight (for desflurane, 168.04);

D = density of desflurane, 1.4892 at 10°C;

Blood ⁄ gas partition coefficient (desflurane) = 0.38 ± 0.01 (0.37–0.39);

According to the ideal gas law, one mole desflurane (molecular weight = 168.04 gram) is equal to 22.4 L of desflurane vapor at 1 atmospheric pressure and 0°C. Therefore, 1 mL desflurane vapor = 7.50 mg. As temperature is increased from 0 to 37°C, 1 mL desflurane vapor should be equal to 6.61 mg, according to the ideal gas law:

The piglet's blood/gas partition coefficient is obtained in the above paragraph. For example, if $\lambda b/g$ is assumed to be 0.38, an equilibrated sample of 100 mL desflurane gas and 100 mL of blood (each at 1% partial pressure) would contain 6.61 mg and 2.51 mg of desflurane, respectively at 37°C (1 atmospheric pressure). Therefore, it can be calculated that 1 mL of 1% desflurane in blood would contain 0.0251 mg of desflurane. In other words, the gas chromatogram count measured from the headspace was registered as containing 1% of desflurane in blood, after 0.0251 mg of desflurane was injected into a 10 mL vial and equilibrated with 1 mL of blank blood.

V. Calibration curve for measuring blood desflurane concentrations

A bottle of desflurane was immersed in a water bath at 4°C for 1 hour before use. Five known amounts of desflurane liquid were drawn up using a microsyringe (Hamilton 0.5 µL syringe; No. 86259) and injected into five 10-mL glass vials. Each vial contained one mL of the pig's blank blood at 4°C, and was allowed to equilibrate for 30 minutes. A linear relationship between peak desflurane counts (y-axis) and desflurane concentrations (x-axis) was established with the correlation coefficients ranging between 0.9975 and 0.9998. The analytical range for the desflurane concentration was 0.7% to 17.5%. The concentration of desflurane in blood was then determined from this calibration curve. The partial pressure of desflurane in the blood phase was calculated from the calibration curve of a known amount of desflurane. The chromatographic area was proportional to the desflurane partial pressure over the entire range of partial pressures studied. Precision and accuracy were determined on spiked piglet samples at six partial pressures (0.7% to 17.5%) with respect to calibration graph prepared every day. The limit of detection was 0.02 % mL/L gas based on signal-to-noise ratio of 3. The limit of quantification of the method for standard samples was 0.1 % mL/L blood. The precision of the method was expressed as the within-day and between-day coefficient of variation (%) less than 7.4%.
